# Relationship between Baseline [^18^F]FDG PET/CT Semiquantitative Parameters and BRCA Mutational Status and Their Prognostic Role in Patients with Invasive Ductal Breast Carcinoma

**DOI:** 10.3390/tomography8060222

**Published:** 2022-10-27

**Authors:** Francesco Dondi, Domenico Albano, Pietro Bellini, Luca Camoni, Giorgio Treglia, Francesco Bertagna

**Affiliations:** 1 Nuclear Medicine, ASST Spedali Civili di Brescia, 25123 Brescia, Italy; 2 Nuclear Medicine, Università degli Studi di Brescia and ASST Spedali Civili di Brescia, 25123 Brescia, Italy; 3 Nuclear Medicine, Imaging Institute of Southern Switzerland, Ente Ospedaliero Cantonale, 6500 Bellinzona, Switzerland; 4 Department of Nuclear Medicine and Molecular Imaging, Lausanne University Hospital and University of Lausanne, 1011 Lausanne, Switzerland; 5 Faculty of Biomedical Sciences, Università della Svizzera italiana, 6900 Lugano, Switzerland

**Keywords:** PET, positron emission tomography, breast cancer, BRCA, FDG, nuclear medicine

## Abstract

Aim: To assess the relationship between [^18^F]FDG PET/CT, breast cancer gene (BRCA) status, and their prognostic role in patients with ductal breast cancer (DBC). Methods: Forty-one women were included. PET/CT semiquantitative parameters such as standardized uptake value (SUV) body weight max (SUVmax), SUV body weight mean (SUVmean), SUV lean body mass (SUVlbm), SUV body surface area (SUVbsa), metabolic tumor volume (MTV), total lesion glycolysis (TLG), ratio SUVmax/blood-pool (S-BP), and ratio SUVmax/liver (S-L) were also extracted. The relationship between these parameters, BRCA, and other clinicopathological features were evaluated. Kaplan–Meier, univariate, and multivariate analyses were performed to find independent prognosticators for progression free (PFS) and overall survival (OS). Results: Significant positive correlations between BRCA status and SUVmax (*p*-value 0.025), SUVlbm (*p*-value 0.016), and SUVbsa (*p*-value 0.018) were reported. Mean PFS was 53.90 months with relapse/progression of disease occurring in nine (22.0%) patients; mean OS was 57.48 months with death occurring in two (4.9%) patients. Survival curves revealed TLG, MTV, and BRCA status as prognosticator for PFS; BRCA was also a prognosticator for OS. Univariate and multivariate analyses did not confirm such insights. Conclusion: We reported a correlation between some PET/CT parameters and BRCA status; some insights on their prognostic role have been underlined.

## 1. Introduction

Breast cancer (BC) is the most common form of cancer in women [1,2]. In general, developed countries have higher rates of BC cases, but, despite that, BC is the most common cause of cancer mortality in women in developing countries [2]. BC patients with locally advanced neoplasms or metastatic diseases have in general poor prognoses [1]. The presentation of BC can be very heterogeneous, varying from asymptomatic forms to the presence of pain or palpable masses [3].

Many risk factors related to the development of BC have been proposed [4]. In particular, hormonal mediated risk factors, environmental factors, and genetic risk factors have been targeted as able to give birth to BC [2]. Between genetic risk factors, mutations to the breast cancer gene (BRCA) (BRCA1 and BRCA2) are thought to be able to give approximately from 5% to 10% of all BC [2]. These genes have the function to repair DNA double-strand breaks by homologous recombination, and it has been reported that their mutation can correspond to a 10-fold increase in BC [5]. In this setting, an increase in the risk to develop contralateral second primary BC in patients with BRCA mutations has been reported [6]. Furthermore, the probability of subjects with a mutation in BRCA1 and BRCA2 genes to develop BC in their lifetime is 57–65% with a 45–49% probability [7]. The prognostic role of BRCA mutations is, however, still controversial with heterogeneous evidence in the literature [8,9,10,11].

The radiological diagnosis of BC is based on the use of various imaging techniques including mammography, ultrasound (US), computed tomography (CT), and nuclear magnetic resonance spectroscopy (MR) [12]. In the last decade, the role of positron emission tomography/CT (PET/CT) with various tracers and, in particular, fluorine-18 fluorodeoxyglucose ([^18^F]FDG) for the assessment of a great number of neoplastic and non-neoplastic diseases is increasing, and the thoracic district does not make an exception [13,14,15,16,17]. In this setting, the role of this hybrid imaging modality for the staging and restaging of BC has been proven, given its ability to evaluate the whole body [18]. Furthermore, there are growing evidences that support the role of baseline [^18^F]FDG PET/CT in the risk stratification of advanced BC and the role of semiquantitative parameters, such as standardized uptake value (SUV), to predict outcomes of BC patients [19].

Recently, a strong correlation between [^18^F]FDG PET/CT and BRCA status has been underlined [20]. The aim of our study is, therefore, to confirm the correlation between baseline [^18^F]FDG PET/CT and BRCA in patients with invasive ductal BC (DBC) and to assess the prognostic role of these two factors.

## 2. Materials and Methods

### 2.1. Patients Selection

Our institutional database was retrospectively screened in order to find patients submitted to our center to perform [^18^F]FDG PET/CT for the initial staging of BC. The screening was performed from January 2016 to March 2022 and a total of 1070 women were extracted. Inclusion criteria were: the presence of a histological proven diagnosis of DBC, the presence of a baseline [^18^F]FDG PET/CT performed before any treatment demonstrating tracer uptake, and a genetic evaluation assessing the presence of mutation of BRCA1 and -2 genes. The presence of BRCA mutations was assessed with next generation sequencing. As a consequence, after applying these inclusion criteria, 41 patients were included in the present study.

For each patient, clinical and pathological information about age, size of BC, TNM category, American Joint Commission on Cancer (AJCC) VIII Edition stage, histological classification, grading, human epidermal growth factor receptor 2 (HER2) status, Ki-67 expression, estrogen receptor (ER) expression, progesterone receptor (PR) expression, and therapy performed after the initial evaluations were collected.

### 2.2. The [^18^F]FDG PET/CT Acquisition and Interpretation

In order to perform [^18^F]FDG PET/CT scan, 3.5–4.5 MBq/kg of [^18^F]FDG was intravenously injected to the patients. All the subjects fasted for at least 6 h before the administration of the tracer, the mean blood glucose level was 106 mg/dL (range: 82–148, standard deviation [SD]: 18), and before images acquisition patients were instructed to void. No contrast agent was administered and no intestinal preparation was used. Scans were performed 60 min after [^18^F]FDG injection, from the vertex to the midthigh on a Discovery ST or a Discovery 690 PET/CT tomograph (General Electric Company, GE, Milwaukee, Wisconsin, USA) with standard parameters (CT: 80 mA, 120 kV; PET: 2.5–4 min per bed position, PET step of 15 cm). Images were reconstructed with a 256 × 256 matrix and a 60-centimeter field of view. On Discovery 690 tomograph, time of flight (TOF) and point spread function (PSF) algorithm were used for the reconstruction of images, with filter cut-off 5 mm, 18 subsets, and 3 iterations. For Discovery ST tomograph, an ordered subset expectation maximization (OSEM) algorithm, with filter cut-off 5 mm, 21 subsets, and 2 iterations, was applied.

Two experienced nuclear medicine physicians visually and semiquantitatively analyzed the PET/CT images by consensus. Every focal tracer uptake deviating from physiological distribution and higher than the background was regarded as suggestive of disease localization. The semiquantitative analysis of images was performed by measuring maximum standardized uptake value (SUV) body weight (SUVmax), mean SUV body weight (SUVmean), SUV lean body mass (SUVlbm), and SUV body surface area (SUVbsa) of all hypermetabolic lesions with the use of a round-shaped volume of interest (VOI) with 10 millimeters diameter, placed in the point of higher tracer uptake. The SUVmax of liver and blood-pool were used to calculate a ratio between SUVmax of the lesions and these two parameters (S-L and S-BP, respectively). In this setting, the SUVmax of the liver was calculated at the VIII hepatic segment from transaxial PET images using a round-shaped 10-millimeter volume of interest (VOI). The SUVmax of the blood-pool was calculated at the aortic arch by using transaxial PET images with a round-shaped VOI with 10 millimeters diameter, not involving the vessel wall.

In order to measure MTV from attenuation-corrected [^18^F]FDG PET/CT images, SUV-based automated contouring program (Advantage Workstation 4.6, GE Healthcare, Milwaukee, WI, USA) was applied, using an iso-contouring threshold method based on 41% of the SUVmax, as recommended by the European Association of Nuclear Medicine [21]. Furthermore, TLG was calculated as the sum of the product of MTV of each lesion and its SUVmean. Nodal and distant metastasis uptakes were comprised in the extraction of volumetric parameters only if the presence of disease was proven by histological evaluation or other imaging modalities.

### 2.3. Statistical Analysis

All statistical analyses were performed using MedCalc Software version 18.1 for Windows (Ostend, Belgium). The descriptive analysis of categorical variables comprised the calculation of simple and relative frequencies. The numeric variables were described as mean, SD, minimum, and maximum (range). T-test, Kruskall–Wallis, and Anova tests were used to assess the correlation between the aforementioned PET/CT semiquantitative parameters and the clinicopathological characteristics of patients. Furthermore, Chi-square test was applied to evaluate the correlation between BRCA mutations and clinicopathological features. For all analyses, a *p*-value <0.05 was considered statistically significant.

### 2.4. Survival Analysis

In order to estimate the survival rate and the risk of disease progression, overall survival (OS) and progression free survival (PFS) were calculated. In this setting, OS was defined as the time in months from the date of baseline [^18^F]FDG PET/CT to the date of death for any cause or to the date of last follow-up. Furthermore, PFS was calculated as the time in months between baseline [^18^F]FDG PET/CT scan and the date of first documented relapse or progression of disease, based on radiological imaging and/or biopsy results.

Kaplan–Meier analysis was used to draw survival curves and log-rank test was used to compare such curves. In order to perform the aforementioned analysis, PET/CT semiquantitative parameters were dichotomized based on their median value. Furthermore, Cox regression model was applied to identify independent prognosticators between clinicopathological and PET/CT features. In this setting, PET/CT semiquantitative parameters, age, size, percentage of Ki-67, and percentage of ER expression were again dichotomized based on median value. The decision to dichotomize such parameters based on their median value was arbitrarily taken based on the advice of our statistician, given the fact that this method divides the population in two groups with identical numerosity. The percentage of PR expression was dichotomized by using the reference value of 50%. Furthermore, stage was dichotomized between stage I–II and III–IV, grading was dichotomized between grade 2 and grade 3, while therapy was dichotomized based on the presence or the absence of radiotherapy (RT) in the therapeutic algorithm. Dichotomization based on the presence of nodal metastasis, the presence of BRCA1 or BRCA2 mutations, and histological classification were also considered in these analyses. In this setting, estimates of the predictive effect for PFS and OS were expressed as hazard ratios (HRs) in univariate and multivariate Cox regression analyses with a 95% confidence interval (CI).

## 3. Results

### 3.1. Patients Characteristics

The mean age of our cohort was 45 years (SD 13, range 23–86) (Table 1). The most part of the neoplasms was localized in the left breast (24 patients, 58.5%), compared to the right breast (17 subjects, 41.5%). The mean size of the lesions, histologically measured, was 27.7 mm (mm) (12.0, 10.0–81.0); G2 disease was present in 12 patients (29.3%); 29 subjects (70.7%) had a G3 disease.

Regarding BRCA mutation, twenty-eight subjects (68.3%) did not demonstrate any gene alterations while thirteen patients (31.7%) had a mutation in these genes. In particular, BRCA1 mutation was present in seven patients (17.1%), while six subjects (14.6%) had BRCA2 mutation. Furthermore, the mean Ki-67 percentage of expression was 46.83% (25.6, 5.0–98.0), the mean percentage of expression of ER was 52.29% (46.1, 0.0–100.0), and the mean percentage of expression of PR was 19.85% (31.9, 0.0–98.0). Regarding HER2 expression status, twenty-seven patients (65.9%) were negative while fourteen (34.1%) were positive.

Speaking about disease stage, seven patients (17.1%) had a stage I disease, seventeen patients (41.5%) had a stage II disease, eight patients (19.5%) had a stage III disease, while stage IV disease was present in nine patients (21.9%).

### 3.2. The ^18^F-FDG PET/CT Results

**Figure 1:**
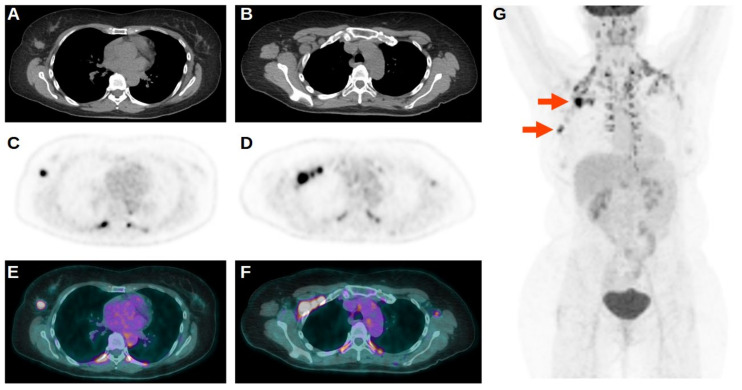
Axial CT (**A**,**B**), axial PET (**C**,**D**), axial fused PET/CT, (**E**,**F**) and maximum intensity projection (MIP) (**G**) of a [^18^F]FDG PET/CT scan performed in a patient positive for BRCA1 mutation, demonstrating intense uptake of tracer on primary right BC and concomitant multiple focal uptakes on metastatic axillary and retropectoral nodes (red arrows on MIP). Brown adipose tissue activation was also reported.

At baseline evaluation with [^18^F]FDG PET/CT, all scans were positive on primary DBC site. Furthermore, the presence of BC nodal metastases was reported in twenty-five subjects (61.0%), while the remaining sixteen patients (39.0%) were negative for nodal localization. In this setting, all twenty-five patients with positive nodes had the presence of at least one axillary lymph node involved and, furthermore, one of them had also retropectoral nodal localization of disease, while two subjects had both retropectoral and internal mammary nodes localization (Figure 1). Distant metastases were present in two patients (4.9%): one subject had the presence of multiple liver localization of BC while the other subject had multiple skeletal localization. As a consequence, the remaining thirty-nine patients (95.1%) did not demonstrate the presence of distant metastasis.

After baseline evaluation of disease with [^18^F]FDG PET/CT, all patients were treated according to the therapeutic algorithm specific for their disease stage and all of them performed surgery. Furthermore, six patients (14.6%) were also treated with chemotherapy (ChT), ten patients (24.4%) with ChT and RT, four patients (9.8%) with ChT, RT, and immunotherapy (IT), four patients (9.8%) with ChT, RT, IT, and hormonal therapy (HT), four patients (9.8%) with ChT, RT, and HT, two patients (4.9%) with ChT and IT, five patients (12.1%) with ChT, IT, and HT, and six patients (14.6%) with ChT and HT.

The mean PFS of our cohort was 53.90 months (30.24, 3.15–150.98) and relapse or progression of disease was experienced by nine patients (22.0%). In particular, two subjects developed multiple nodal localization BC, two patients had progression on both the liver and skeleton, one patient had progression on the liver, lymph nodes, and skeleton, one patient had only bone metastasis, one patient had progression only on the liver, one patient experienced the presence of multiple lung localization, and one patient had both local relapse and concomitant nodal metastasis. The mean OS was 57.48 months (25.91, 10.63–150.98), and death occurred only in two patients (4.3%).

The statistical analysis revealed a correlation between MTV and TLG with stage of disease (*p*-value 0.006 and 0.003, respectively), while the size of the primary DBC had significant correlation with TLG (*p*-value 0.038). Furthermore, we reported a positive correlation between MTV (*p*-value 0.002) and TLG (*p*-value 0.014) with the presence of nodal metastases revealed. Regarding BRCA status, significant correlations were found with SUVmax (*p*-value 0.025), SUVlbm (*p*-value 0.016), and SUVbsa (*p*-value 0.018). Instead, a correlation between Ki-67 expression with SUVmax (*p*-value 0.002), SUVmean (*p*-value 0.003), SUVlbm (*p*-value 0.004), SUVbsa (*p*-value 0.020), S-L (*p*-value 0.002), and S-BP (*p*-value 0.006) was underlined. Speaking about hormonal receptors, both ER and PR expressions were significantly correlated with SUVmax (*p*-value 0.007 and 0.022, respectively), SUVlbm (*p*-value 0.004 and 0.025, respectively), SUVbsa (*p*-value 0.003 and 0.023, respectively), S-L (*p*-value 0.012 and 0.033, respectively), and S-BP (*p*-value 0.017 and 0.029, respectively). Furthermore, SUVmean (*p*-value 0.015) had a significant correlation with ER expression. No significant correlations were reported for all other combinations between clinicopathological features and PET/CT semiquantitative parameters (Table 2).

Correlations between clinicopathological features and BRCA status were also investigated, reporting only a statistically significant value for the presence of nodal metastases status (*p*-value 0.034) (Table 3).

### 3.3. Prognostic Value of ^18^F-FDG PET/CT

**Figure 2:**
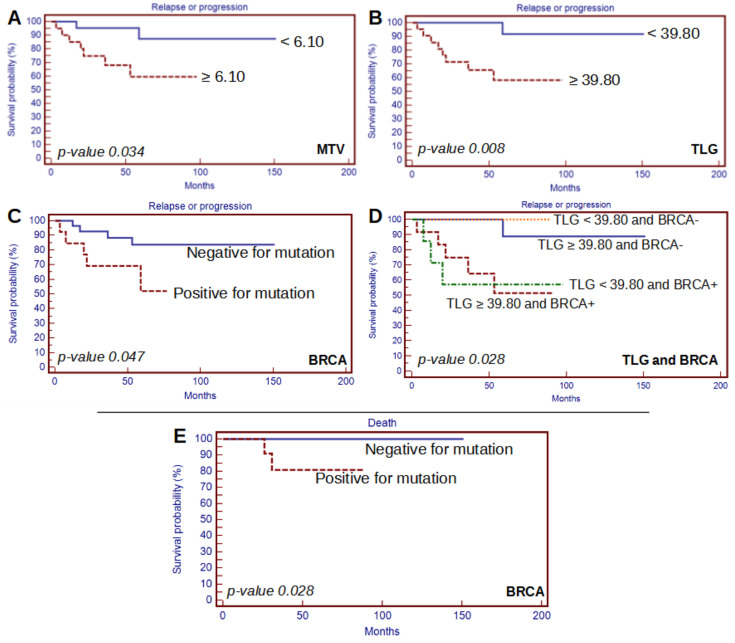
Significant survival curve analysis for MTV (**A**), TLG (**B**), BRCA status, (**C**) and TLG combined with BRCA status (**D**) regarding PFS and BRCA status regarding OS (**E**).

Kaplan–Meier analysis performed for PET/CT semiquantitative parameters and BRCA status revealed MTV, TLG, and BRCA status as predictors for PFS (*p*-value 0.034, 0.008, and 0.047, respectively); BRCA status was also underlined as a prognosticator for OS (*p*-value 0.024). Furthermore, the same analysis was performed also considering both semiquantitative parameters and BRCA status together. In this setting, only the specific analyses performed with BRCA and TLG were reported as statistically significant for PFS (*p*-value 0.028), and we reported no significant prognosticators for OS (Table 4). In particular, patients with both BRCA mutations and high TLG values had lower PFS compared to patients with one of these characteristics or none of them (Figure 2).

Univariate analysis with all aforementioned clinicopathological and [^18^F]FDG PET/CT semiquantitative parameters revealed stage (*p*-value 0.012) and TLG (*p*-value 0.024) as affordable prognosticators for PFS; however, subsequent multivariate analysis did not confirm any of the parameters as independent prognostic factors. For what concerns OS, univariate and subsequent multivariate analyses did not reveal any independent prognostic factors (Table 5).

## 4. Discussion

The main aim of this manuscript was to correlate [^18^F]FDG PET/CT semiquantitative parameters and BRCA mutational status, and in this setting, a strong correlation between such parameters has been underlined by our results, in particular for SUVmax, SUVlbm, and SUVbsa. Our findings confirm the insights proposed by Özdemir et al. [20], who underlined that SUVmax of the primary breast lesion in patients with BC was higher in subjects with BRCA1 or -2 mutations, compared to patients without such mutations. In this setting, we underlined a relationship not only for SUVmax, giving more strength to previous findings. In particular, one of the strength points of our work is the fact that we focused our analyses on DBC; therefore, our cohort was relatively limited but homogeneous in terms of histology.

When evaluating the correlation between [^18^F]FDG PET/CT semiquantitative parameters and clinicopathological features, we reported a strong correlation between MTV and TLG with stage and size of primary BC. These results are not surprising, considering that such parameters reflect the volumetric characteristics of the disease. Furthermore, our data and analysis revealed a strong correlation between MTV and TLG with the presence of nodal metastases. Our findings confirm what was underlined by Mori et al. [22], that reported [^18^F]FDG PET/CT showed high diagnostic performance for N-staging in BC patients, especially a high negative predictive value. Similar findings were also reported when considering dual-phase imaging [23].

Özdemir et al. [20] also reported a strong correlation between BRCA mutational status and some clinicopathological features of BC. We demonstrated that patients positive for BRCA mutations had higher proportion of nodal metastasis at [^18^F]FDG PET/CT. When considering hormonal receptors, both ER and PR expression were significantly correlated with SUVmax, SUVlbm, SUVbsa, S-L, and S-BP, while ER expression was correlated with SUVmean. In this setting, the correlation between [^18^F]FDG PET/CT semiquantitative parameters and ER and PR expression in BC has been widely evaluated in the literature, with heterogeneous results [24,25].

In general, prognostic analysis reported some insights on the role of [^18^F]FDG PET/CT and BRCA status, in particular for PFS. Survival curve analyses, by combining both BRCA mutational status and PET/CT semiquantitative parameters, revealed that patients with both BRCA mutations and high TLG values had lower PFS compared to patients with one of these characteristics or none of them. Furthermore, when considering only BRCA mutational status, Kaplan–Meier analysis revealed its prognostic role for PFS and OS; however, this was not confirmed with univariate and multivariate analyses. The effect of BRCA mutations on BC prognosis has been investigated in the literature with controversial results; however, a meta-analysis by Baretta et al. [26] suggested to perform the evaluation of BRCA mutational status in patients with a high risk of harboring BRCA germline mutations to better define the prognosis of BC in these patients. Interestingly, this analysis was conducted also by considering the difference between BRCA1 and BRCA2; unfortunately, in our work we were not able to perform such analyses given the small sample of patients in each category.

As mentioned, for what concerns [^18^F]FDG PET/CT parameters, Kaplan–Meier analysis revealed MTV and TLG as prognosticators for PFS and TLG as prognosticators for OS. In this setting, we reported a strong positive correlation between such parameters and stage or size of disease. In this setting, after univariate analysis for PFS, TLG together with stage of disease were confirmed as independent prognostic factors, but, despite that, multivariate analysis did not confirm any independent prognostic factor. Univariate and multivariate analyses for OS did not report any affordable prognosticators. Considering all the aforementioned data, the prognostic role of [^18^F]FDG PET/CT semiquantitative parameters cannot be clearly confirmed in our cohort.

The prognostic role of baseline [^18^F]FDG PET/CT in BC has been widely investigated in the literature. In particular, despite the high heterogeneity of characteristics of the included studies, a review by Caresia Aroztegui et al. [26] concluded that [^18^F]FDG PET/CT has prognostic value in BC. In particular, its great therapeutic impact in locally advanced BC has been demonstrated, revealing its usefulness in two ways: by detecting distant metastases occult to other techniques and by detecting glycolytic activity in the primary tumor and axillary lymph nodes [26]. Similarly, a recent meta-analysis, focused on the prognostic role of volumetric parameters such as MTV and TLG, underlined that they were significant prognostic factors for outcome in patients with BC. In particular, BC patients with a high MTV or TLG from primary tumor had a higher risk of adverse events [27].

Our work is not without limitations, starting from the retrospective design of the study. The presence of heterogeneous types of treatment after primary tumor evaluation is another potential confounding factor. Furthermore, as mentioned, our cohort was relatively limited but homogeneous in terms of histology. In this setting, our population is highly selected since genetic tests are not routinely performed in all BC patients and, therefore, the lack of generalizability of our work has to be taken into account. Further and multicentric studies are suggested to strengthen our assumptions.

## 5. Conclusions

We demonstrated the correlation between some baseline [^18^F]FDG PET/CT semiquantitative parameters and the mutational status of BRCA in patients with DBC. No clear evidences of their prognostic role have been clearly demonstrated.

## Figures and Tables

**Table 1:** Characteristics of the 46 patients included in the study.

Characteristic	n (%)
Age (mean ± SD, range)	45 ± 13, 23–86
Size (mean ± SD, range) (mm)	27.7 ± 12.8, 10.0–81.0
Grading	
G2	12 (29.3%)
G3	29 (70.7%)
Breast	
Left	24 (58.5%)
Right	17 (41.5%)
BRCA mutation	
No	28 (68.3%)
BRCA1	7 (17.1%)
BRCA2	6 (14.6%)
Ki-67 expression (mean ± SD, range) (%)	46.83 ± 25.6, 5.0–98.0
ER expression (mean ± SD, range) (%)	52.29 ± 46.1, 0.0–100.0
PR expression (mean ± SD, range) (%)	19.85 ± 31.9; 0.0–98.0
HER2 status	
Negative	27 (65.9%)
Positive	14 (34.1%)
AJCC stage	
I	7 (17.1%)
II	17 (41.5%)
III	8 (19.5%)
IV	9 (21.9%)
Nodal metastasis	
Yes	25 (61.0%)
No	16 (39.0%)
Distant metastasis	
Yes	2 (4.9%)
No	39 (95.1%)
Therapy	
Surgery, ChT	6 (14.6%)
Surgery, ChT, RT	10 (24.4%)
Surgery, ChT, RT, IT	4 (9.8%)
Surgery, ChT, RT, IT, OT	4 (9.8%)
Surgery, ChT, RT, OT	4 (9.8%)
Surgery, ChT, IT	2 (4.9%)
Surgery, ChT, IT, OT	5 (12.1%)
Surgery, ChT, OT	6 (14.6%)
PET/CT parameters	
SUVmax (mean ± SD, range)	9.19 ± 6.15, 1.74–27.88
SUVmean (mean ± SD, range)	5.43 ± 3.91, 1.02–18.55
SUVlbm (mean ± SD, range)	6.77 ± 4.67, 1.22–19.53
SUVbsa (mean ± SD, range)	2.57 ± 1.73, 0.47–7.17
S-L (mean ± SD, range)	3.65 ± 2.29, 0.73–8.77
S-BP (mean ± SD, range)	4.68 ± 3.21, 0.87–14.28
MTV (mean ± SD, range)	9.46 ± 9.28, 1.30–42.10
TLG (mean ± SD, range)	70.52 ± 90.36, 5.70–463.40
Relapse or progression	
Yes	9 (22.0%)
No	32 (78.0%)
Death	
Yes	2 (4.9%)
No	39 (95.1%)
PFS months (mean ± SD, range)	53.90 ± 30.24, 3.15–150.98
OS months (mean ± SD, range)	57.48 ± 25.91, 10.63–150.98

SD: standard deviation; mm: millimeters; BRCA: breast cancer gene; ER: estrogen receptor; PR: progesterone receptor; HER2: human epidermal growth factor receptor 2; AJCC: American Joint Commission on Cancer; PET/CT: positron emission tomography/computed tomography; ChT: chemotherapy; RT: radiotherapy; IT: immunotherapy; HT: hormonal therapy; SUVmax: standardized uptake value body weight maximum; SUVmean: standardized uptake value body weight mean; SUVlbm: standardized uptake value lean body mass; SUVbsa: standardized uptake value body surface area; S-BP: SUVmax/blood-pool uptake; S-L: SUVmax/liver uptake; MTV: metabolic tumor volume; TLG: total lesion glycolysis; OS: overall survival; PFS: progression free survival.

**Table 2:** Correlation between clinicopathological and baseline [^18^F]FDG PET/CT semiquantitative parameters.

	SUVmax	*p*-Value	SUVmean	*p*-Value	SUVlbm	*p*-Value	SUVbsa	*p*-Value	S-L	*p*-Value	S-BP	*p*-Value	MTV	*p*-Value	TLG	*p*-Value
AJCC Stage		*0.463*		*0.379*		*0.367*		*0.391*		*0.368*		*0.324*		*0.006*		*0.008*
I	11.60		5.11		8.99		3.42		4.39		5.08		2.22		15.73	
II	7.71		4.26		5.37		2.09		2.92		3.81		7.13		53.36	
III	10.03		6.24		7.23		2.68		3.78		4.42		13.10		90.47	
IV	10.70		7.16		7.24		2.71		4.32		6.24		16.24		124.06	
Age		*0.522*		*0.449*		*0.309*		*0.396*		*0.273*		*0.334*		*0.531*		*0.334*
<44	10.38		5.90		7.41		2.79		4.17		5.45		8.83		76.88	
≥44	8.64		4.98		6.15		2.36		3.15		3.94		10.06		64.47	
HER2 status		*0.371*		*0.741*		*0.509*		*0.386*		*0.364*		*0.296*		*0.610*		*0.923*
Negative	10.26		5.81		7.38		2.81		3.96		5.07		8.83		75.17	
Positive	7.99		4.68		5.58		2.09		3.04		3.92		10.67		61.55	
Size (mm)		*0.814*		*0.597*		*0.753*		*0.834*		*0.513*		*0.916*		*0.067*		*0.025*
<26	9.32		4.96		6.90		2.61		3.84		4.58		6.61		36.96	
≥26	9.63		5.84		6.65		2.53		3.48		4.77		11.92		99.51	
Grading		*0.491*		*0.430*		*0.731*		*0.709*		*0.406*		*0.315*		*0.351*		*0.240*
2	8.43		5.09		6.19		2.32		3.11		3.89		8.50		75.20	
3	9.92		5.57		7.01		2.67		3.87		5.01		9.85		68.59	
N+		*0.168*		*0.336*		*0.309*		*0.181*		*0.* *057*		*0.* *051*		*0.002*		*0.014*
Yes	10.23		5.36		7.31		2.85		4.25		5.35		12.87		97.61	
No	9.06		5.47		6.42		2.39		3.26		4.25		4.25		28.20	
BRCA mutation		*0.025*		*0.154*		*0.016*		*0.018*		*0.* *058*		*0.278*		*0.338*		*0.* *069*
Yes	12.60		6.71		9.30		3.49		4.64		5.49		11.52		108.00	
No	8.04		4.83		5.59		2.14		3.19		4.30		8.50		53.12	
Ki-67 status		*0.002*		*0.003*		*0.003*		*0.002*		*0.002*		*0.006*		*0.960*		*0.190*
<40	6.79		3.75		4.75		1.80		2.64		3.39		9.51		52.33	
≥40	12.32		7.19		8.88		3.38		4.71		6.03		9.40		89.62	
ER status		*0.007*		*0.015*		*0.004*		*0.003*		*0.012*		*0.017*		*0.300*		*0.653*
<80	7.03		4.01		4.82		1.82		2.79		3.53		7.90		64.23	
≥80	12.07		6.92		8.81		3.32		4.55		5.88		10.94		77.14	
PR status		*0.022*		*0.050*		*0.025*		*0.023*		*0.033*		*0.029*		*0.185*		*0.749*
<50	4.25		2.56		2.87		1.10		1.83		2.07		8.66		59.43	
≥50	10.38		5.94		7.43		2.82		3.96		5.13		14.13		72.42	
M+		*0.275*		*0.301*		*0.453*		*0.439*		*0.142*		*0.065*		*0.691*		*0.319*
Yes	14.17		8.25		9.23		3.51		5.98		8.74		12.05		133.35	
No	9.25		5.28		6.64		2.52		3.53		4.47		9.32		67.30	

N+: presence of nodal metastasis; M+: presence of distant metastasis; BRCA: breast cancer gene; ER: estrogen receptor; PR: progesterone receptor; HER2: human epidermal growth factor receptor 2; AJCC: American Joint Commission on Cancer; PET/CT: positron emission tomography/computed tomography; SUVmax: standardized uptake value body weight maximum; SUVmean: standardized uptake value body weight mean; SUVlbm: standardized uptake value lean body mass; SUVbsa: standardized uptake value body surface area; S-BP: SUVmax/blood-pool uptake; S-L: SUVmax/liver uptake; MTV: metabolic tumor volume; TLG: total lesion glycolysis.

**Table 3:** Correlation between clinicopathological parameters and BRCA status.

	BRCA without Mutation	BRCA with Mutation	*p*-*Value*
AJCC Stage			*0.218*
I	5 (12.2%)	2 (4.9%)	
II	13 (31.7%)	4 (9.7%)	
III	3 (7.3%)	5 (12.2%)	
IV	7 (17.1%)	2 (4.9%)	
Age			*0.053*
<42	14 (34.1%)	6 (14.7%)	
≥42	14 (34.1%)	7 (17.1%)	
HER2 status			*0.084*
Negative	16 (39.0%)	11 (26.8%)	
Positive	12 (29.3%)	2 (4.9%)	
Size (mm)			*0.173*
<26	15 (36.6%)	4 (9.7%)	
≥26	13 (31.7%)	9 (22.0%)	
Grading			*0.552*
2	9 (22.0%)	3 (7.3%)	
3	19 (46.3%)	10 (24.4%)	
N+			*0.034*
Yes	14 (34.1%)	11 (26.8%)	
No	14 (34.1%)	2 (4.9%)	
Ki67 status			*0.265*
<41	16 (39.0%)	5 (12.2%)	
≥41	12 (29.3%)	8 (19.5%)	
ER status			*0.818*
<50	14 (34.1%)	6 (14.7%)	
≥50	14 (34.1%)	7 (17.1%)	
PR status			*0.391*
<50	23 (56.1%)	12 (29.3%)	
≥50	5 (12.2%)	1 (2.4%)	
M+			*0.322*
Yes	2 (4.9%)	0 (0.0%)	
No	26 (63.4%)	13 (31.7%)	

N+: presence of nodal metastasis; M+: presence of distant metastasis; BRCA: breast cancer gene; ER: estrogen receptor; PR: progesterone receptor; HER2: human epidermal growth factor receptor 2; AJCC: American Joint Commission on Cancer.

**Table 4:** Kaplan–Meier results (*p*-value) for PFS and OS.

	Single Analysis		BRCA and Semiquantitative Parameters	
	PFS	OS	PFS	OS
SUVmax	*0.520*	*0.* *854*	*0.161*	*0.174*
SUVmean	*0.* *545*	*0.854*	*0.366*	*0.288*
SUVlbm	*0.977*	*0.854*	*0.376*	*0.174*
SUVbsa	*0.977*	*0.854*	*0.376*	*0.174*
S-L	*0.947*	*0.854*	*0.388*	*0.174*
S-BP	*0.806*	*0.9* *19*	*0.707*	*0.319*
MTV	*0.0* *34*	*0.117*	*0.199*	*0.479*
TLG	*0.008*	*0.1* *51*	*0.028*	*0.438*
BRCA	*0.* *047*	*0.* *024*		

PFS: progression free survival; OS: overall survival; SUVmax: standardized uptake value body weight maximum; SUVmean: standardized uptake value body weight mean; SUVlbm: standardized uptake value lean body mass; SUVbsa: standardized uptake value body surface area; S-BP: SUVmax/blood-pool uptake; S-L: SUVmax/liver uptake; MTV: metabolic tumor volume; TLG: total lesion glycolysis; BRCA: breast cancer gene.

**Table 5:** Univariate and multivariate analyses with clinicopathological and [^18^F]FDG PET/CT semiquantitative parameters for PFS and OS.

	Univariate Analysis		Multivariate Analysis	
	*p-Value*	HR (95% CI)	*p-Value*	HR (95% CI)
PFS				
BRCA status	*0.797*	1.19 (0.30–4.76)		
Age	*0.858*	1.12 (0.30–4.19)		
Stage	*0.012*	14.45 (1.81–114.87)	*0.059*	8.09 (0.92–70.49)
Size	*0.386*	1.84 (0.46–7.32)		
Grading	*0.678*	1.39 (0.29–6.66)		
HER2 status	*0.390*	0.50 (0.11–2.40)		
Ki-67 expression	*0.563*	1.47 (0.39–5.46)		
ER expression	*0.862*	1.12 (0.30–4.16)		
PR expression	*0.557*	1.60 (0.33–7.67)		
Therapy	*0.121*	3.46 (0.72–16.53)		
N+	*0.107*	5.52 (0.69–43.73)		
SUVmax	*0.523*	1.53 (0.41–5.70)		
SUVmean	*0.548*	1.49 (0.40–5.55)		
SUVlbm	*0.977*	0.98 (0.26–3.64)		
SUVbsa	*0.977*	0.98 (0.26–3.64)		
S-L	*0.947*	0.95 (0.26–3.54)		
S-BP	*0.806*	0.84 (0.23–3.14)		
MTV	*0.053*	4.73 (0.98–22.75)		
TLG	*0.024*	10.87 (1.36–86.32)	*0.152*	4.94 (0.56–43.63)
OS				
BRCA status	*0.668*	1.83 (0.12–28.94)		
Age	*0.850*	0.76 (0.05–12.16)		
Stage	*0.949*	0.82 (0.34–2.92)		
Size	*0.949*	0.80 (0.31–3.78)		
Grading	*0.* *958*	0.95 (0.12–3.68)		
HER2 status	*0.* *957*	0.99 (0.24–3.07)		
Ki-67 expression	*0.982*	0.97 (0.06–15.29)		
ER expression	*0.953*	0.92 (0.06–14.51)		
PR expression	*0.968*	0.12 (0.01–2.07)		
Therapy	*0.* *949*	0.80 (0.41–3.65)		
N+	*0.952*	1.21 (0.40–3.92)		
SUVmax	*0.855*	1.29 (0.08–20.42)		
SUVmean	*0.855*	1.29 (0.08–20.42)		
SUVlbm	*0.855*	1.29 (0.08–20.42)		
SUVbsa	*0.855*	1.29 (0.08–20.42)		
S-L	*0.855*	1.29 (0.08–20.42)		
S-BP	*0.919*	1.15 (0.07–18.21)		
MTV	*0.948*	0.79 (0.29–2.75)		
TLG	*0.948*	0.79 (0.28–2.75)		

HR: hazard ratio; CI: confidence interval; PFS: progression free survival; OS: overall survival; BRCA: breast cancer gene; ER: estrogen receptor; PR: progesterone receptor; HER2: human epidermal growth factor receptor 2; N+: presence of nodal metastasis; SUVmax: standardized uptake value body weight maximum; SUVmean: standardized uptake value body weight mean; SUVlbm: standardized uptake value lean body mass; SUVbsa: standardized uptake value body surface area; S-BP: SUVmax/blood-pool uptake; S-L: SUVmax/liver uptake; MTV: metabolic tumor volume; TLG: total lesion glycolysis.

## Data Availability

Data available on request due to privacy/ethical restrictions.
